# Expression of human AID in yeast induces mutations in context similar to the context of somatic hypermutation at G-C pairs in immunoglobulin genes

**DOI:** 10.1186/1471-2172-6-10

**Published:** 2005-06-10

**Authors:** Vladimir I Mayorov, Igor B Rogozin, Linda R Adkison, Christin Frahm, Thomas A Kunkel, Youri I Pavlov

**Affiliations:** 1 Mercer University School of Medicine, Macon, GA 31207, USA; 2 National Center for Biotechnology Information NLM, National Institutes of Health, Bethesda MD 20894, USA; 3 Institute of Cytology and Genetics SD RAS, Novosibirsk 630090, Russia; 4 Laboratory of Structural Biology, National Institute of Environmental Health Sciences, National Institutes of Health, Research Triangle Park, NC 27709, USA; 5 Eppley Institute for Research in Cancer, University of Nebraska Medical Center, 986805 Nebraska Medical Center, Omaha, NE 68198, USA; 6 Department of Biochemistry and Molecular Biology, University of Nebraska Medical Center, 986805 Nebraska Medical Center, Omaha, NE 68198, USA; 7 Department of Pathology and Microbiology, University of Nebraska Medical Center, 986805 Nebraska Medical Center, Omaha, NE 68198, USA

## Abstract

**Background:**

Antibody genes are diversified by somatic hypermutation (SHM), gene conversion and class-switch recombination. All three processes are initiated by the activation-induced deaminase (AID). According to a DNA deamination model of SHM, AID converts cytosine to uracil in DNA sequences. The initial deamination of cytosine leads to mutation and recombination in pathways involving replication, DNA mismatch repair and possibly base excision repair. The DNA sequence context of mutation hotspots at G-C pairs during SHM is DGYW/WRCH (G-C is a hotspot position, R = A/G, Y = T/C, W = A/T, D = A/G/T).

**Results:**

To investigate the mechanisms of AID-induced mutagenesis in a model system, we studied the genetic consequences of AID expression in yeast. We constructed a yeast vector with an artificially synthesized human *AID *gene insert using codons common to highly expressed yeast genes. We found that expression of the artificial *hAIDSc *gene was moderately mutagenic in a wild-type strain and highly mutagenic in an *ung1 *uracil-DNA glycosylase-deficient strain. A majority of mutations were at G-C pairs. In the *ung1 *strain, C-G to T-A transitions were found almost exclusively, while a mixture of transitions with 12% transversions was characteristic in the wild-type strain. In the *ung1 *strain mutations that could have originated from deamination of the transcribed stand were found more frequently. In the wild-type strain, the strand bias was reversed. DGYW/WRCH motifs were preferential sites of mutations.

**Conclusion:**

The results are consistent with the hypothesis that AID-mediated deamination of DNA is a major cause of mutations at G-C base pairs in immunoglobulin genes during SHM. The sequence contexts of mutations in yeast induced by AID and those of somatic mutations at G-C pairs in immunoglobulin genes are significantly similar. This indicates that the intrinsic substrate specificity of AID itself is a primary determinant of mutational hotspots at G-C base pairs during SHM.

## Background

The immune system uses several strategies to modify genetic material to generate various types of high affinity antibodies [1]. These strategies enable production of multiple antibody variants to a wide range of different antigens [2]. Initially, antigen receptors are generated by a site-specific recombination process called *V(D)J *recombination occurring in the bone marrow [3]. However, this is not sufficient to assure an adequate immune response. Mature B-lymphocytes migrate to the secondary lymphoid organs where they encounter antigens. Upon activation by antigens, mature B-lymphocytes begin to proliferate and form germinal centers, where immunoglobulin genes undergo additional modifications: class switch recombination (CSR), immunoglobulin gene conversion (IGC) and somatic hypermutation (SHM) [4]. SHM, IGC and CSR, all require active transcription [5] and generate diversity of antibodies, that is followed by selection leading to the production of high affinity antibodies [6]. The frequency of mutations during this process is up to six orders of magnitude higher than in other genes [6]. Most of the mutations are base pair substitutions, occurring with a similar frequency at G-C and A-T base pairs. Statistically preferred hotspots for mutations at G-C pairs are RGYW/WRCY motifs (mutating G-C are underlined, R stands for purine base, Y stands for pyrimidine base and W stands for A or T) [7], or recently refined DGYW/WRCH motifs (D stands for G, T or A) [8]. Hotspots of mutations at A-T pairs are in WA /TW motifs (mutating A-T are underlined) [9].

A major breakthrough in understanding the mechanisms of CSR, IGC and SHM was the discovery that they all depend on activation-induced cytidine deaminase, AID [10-16]. Patients with defective AID have giant germinal centers and elevated levels of only one type of low-affinity antibodies, IgM. They suffer from recurrent bacterial infections in the respiratory tract [17] due to the lack of efficient antibody responses that depend on several crucial steps of B cell terminal differentiation including CSR and SHM. SHM is targeted to specific DNA regions in specialized tissues. Defects in this targeting may result in genome-wide mutagenesis and cancer. B-cell lymphomas possess translocations that bring proto-oncogenes into immunoglobulin loci (see [18]). Constitutive expression of AID in mice leads to an increase of tumor incidence [19].

When discovered, AID was thought to act in mutagenesis and recombination in immunity by RNA editing [10,11,20]. It was proposed that AID edits pre-mRNA encoding a nicking endonuclease that initiates SHM, IGC and CSR [5]. This model is called "RNA-editing" [20]. The AID is homologous to the known RNA-editing enzyme APOBEC1, which deaminates cytosine at position 6666 in ApoB100 mRNA and seemingly has no role in immunity. AID possesses the ability to deaminate cytidine, and shuttles between the nucleus and cytoplasm similar to APOBEC1 [4,5,21,22]. A different hypothesis, called "DNA deamination", suggests AID deaminates cytosine directly and that uracil generated in this reaction triggers downstream reactions leading to genetic instability [23-26] (see [27-33] for reviews).

Experimental evidence is accumulating in favor of the DNA deamination hypothesis of AID function [29,31-34]. AID is able to induce SHM and CSR in hybridomas and in fibroblasts, suggesting that it is the only B-cell specific component required for induction of both genetic events [13,14,35]. AID can also induce mutations when expressed in *E. coli *[24]. These mutations occur in the same DNA sequence motifs as mutations during SHM [8,36]. Therefore, eukaryotic cell-specific components are not necessary for mutagenesis. This mutator effect is enhanced in uracil-DNA glycosylase-deficient *ung1 *strains, which are unable to repair uracil in DNA [37], suggesting that the deamination of cytosine to uracil in DNA is the cause of these mutations [24]. It was found that the expression of two homologous deaminases, APOBEC1 and APOBEC3G, is highly mutagenic in bacteria [38]. Almost all mutations arising upon expression of these deaminases were G-A to A-T transitions, consistent with the DNA deamination model. AID deaminates single-stranded and supercoiled double-stranded DNA [39-44] (see also review [31]). AID exhibits clear DNA sequence context specificity, which resembles the specificity of G-C to the A-T component of SHM mutagenesis (GYW/WRC motifs, see[8,40,44-46]). The specificity of induced mutations in bacteria is consistent with predominant deamination of the non-transcribed DNA strand [36,45], which is thought to be single-stranded during transcription (reviewed in [31]). During SHM, however, both DNA strands are targeted for mutagenesis [7], [47]. This discrepancy between the parameters of SHM in vertebrates and deaminase-induced mutagenesis in prokaryotes still needs to be resolved.

To characterize the initial steps of AID-induced mutations, we examined the specificity of the mutator effect of human AID expressed in yeast. We constructed a yeast vector with an artificially synthesized human *AID *gene insert using codons common to highly expressed yeast genes. We found that expression of the artificial *hAIDSc *gene was moderately mutagenic in the wild-type strain and highly mutagenic in the *ung1 *strain, similar to expression of unmodified human AID [48]. This is consistent with the uracil DNA deamination model of mutagenesis. We identified a spectrum of mutations in the *CAN1 *gene occurring in wild-type and *ung1 *strains expressing *hAIDSc*. We compared the sequence context of AID-induced mutations in yeast at G-C bases with somatic mutations in immunoglobulin genes. These comparisons revealed significantly similar properties and further support the hypothesis that AID is a primary cause of mutations at G-C pairs in immunoglobulin genes during SHM.

## Results

### *hAIDSc *expression and its mutator effect

**Figure 1:**
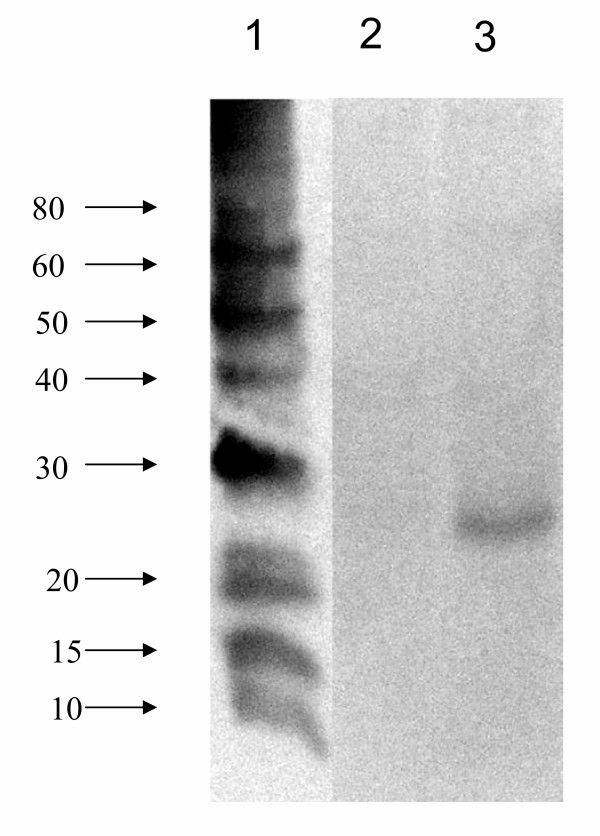
**Western blot analysis of *hAIDSc *expression in yeast**. Yeast strain CG379-3-29RL transformed by expression vector pESC-LEU2 or pESC-LEU-*hAIDSc *were grown to logarithmic phase in a complete minimal medium without leucine. Then cells were washed and transferred into similar medium but containing galactose instead of glucose. Yeast protein extracts were prepared from approximately 200 mg of cells by the glass beads cell disruption method as described in [85]. Proteins were separated using 4–12% gradient PAA NuPage gel (Invitrogen). Transfer to PVDF membrane and reaction with primary antibodies (mouse anti c-myc) and then secondary antibodies (goat antimouse) was accomplished as suggested by the vendor (Western Breeze kit, Invitrogen). Lane 1 – Molecular weight markers (Benchmark, His-Tagged) were detected with antiHisx6 antibodies. Lane 2 – extract of yeast strain containing vector pESC-LEU. Lane 3 – extract of yeast strain containing pESC-LEU-*hAIDSc*.

Codon usage is different in yeast and humans. To improve our system of expression of human *AID *over work published earlier [48], we constructed a new yeast expression vector with the human *AID *gene recoded to use the same codons utilized by highly expressed yeast genes and with a galactose-inducible promoter. Appropriate transformants were grown in galactose-containing medium and the AID protein was readily detected in yeast extracts by Western blot (Fig. 1, lane 3).

The expression of the *hAIDSc *did not result in any profound growth inhibition; the cell titer usually reached 5 × 10^7^, which is typical for galactose-containing minimal medium (data not shown). Mutation rates were analyzed by fluctuation analysis (Table 1). Our strain permits the detection of various classes of genetic events (see Materials and Methods, and also [48,49]). Using this strain we can obtain the express information about the specificity of the mutagenic effect.

The expression of the *hAIDSc *did not induce frameshift mutations to His^+ ^(last column of Table 1). In *Ung1*^+ ^strains, *hAIDSc *expression leads to a 7.6 fold increase in Can^r ^forward mutations and a 3 – 6 fold increase in nonsense mutation reversion (Ade^+^, Trp^+^). The *ung1 *mutation *per se *led to a 5 – 10 fold increase of mutation rates as shown in rows one and four. When the *hAIDSc *was expressed in the *ung1 *strain, the mutator effect was multiplicative for Can^r ^forward mutations (82 fold increase over the wild-type strain) and synergistic for nonsense mutation reversion (a 404 – 1290 fold increase over wild-type). TAG and TAA nonsense mutations cannot revert by true back-mutations via G-C to A-T transitions. We have previously shown by genetic analysis and sequencing of revertants that reversion is caused by dominant suppressors and most likely represent mutations in the anticodon of tRNA genes, which could be G-C to A-Ts [48]. The high response of *ade5-1 *and *trp1-289 *markers to *hAIDSc *may reflect the role of transcription in AID-induced hypermutation in yeast, since tRNA genes are transcribed differently from metabolic genes. The *ura3-29 *allele reversion was stimulated only weakly. It is known, that the allele reverts via various changes at G-C pair in "TCT" DNA sequence context [50], which is different from hotspots of AID deaminations. The results suggest that uracil DNA deamination is the primary source of mutation induced by the *hAIDSc *in yeast and are consistent with our previous studies [48]. Optimized codon usage did not lead to increased mutagenesis under conditions of constant induction of galactose promoter since the mutagenic potential of expression of the *hAIDSc *was comparable with expression of native human *AID *[48].

### Mutagenic specificity of *hAIDSc*

We studied the specificity of mutations in the *CAN1 *gene induced by the expression of the *hAIDSc*. Independent Can^r ^mutants were obtained under conditions of *hAIDSc *expression in the wild-type and the *ung1 *strain. Results of sequencing of mutants are summarized in the Tables 2, 3 and [see Additional file 1]. Most mutations (64 out of 70 in the wild-type and 62 out of 66 in the *ung1 *strain) were at G-C base pairs. Transversions comprised 12% of the mutations at the G-C pairs in wild-type and 1.6% in the *ung1 *strain (Table 4). The decreased proportion of transversions in the *ung1 *strain is consistent with the data obtained earlier in chicken and mice [26,51]. We compared these spectra with the spectra of spontaneous mutations in *CAN1 *in the wild-type strains obtained by Rattrey and coauthors [52], Table 5. The major property of these mutation spectra was a high frequency of frameshift mutations (>20%) [52]. Another feature of the spontaneous mutations is a high frequency of mutations in A-T bases (>50%) and a higher frequency of transversions compared to transitions (>50%) (see also the breakdown of the types of spontaneous mutations obtained previously by other groups [53-55]). These features of *CAN1 *spontaneous mutations are similar to the properties of mutations observed in the yeast *SUP4-o *gene [56]. Thus, the spectra of mutations induced by the expression of the *hAIDSc *are different from spontaneous mutations in yeast genes. This result indicates that spontaneous mutations constitute a minor fraction (if any) of the mutations induced by the expression of the *hAIDSc*.

G-C mutations may arise by putative deamination on the transcribed or non-transcribed DNA strand. Mutations in the *ung1 *strain, representing deamination proclivity without of uracil repair, occur at a higher rate on the transcribed strand (Table 4). This is different from the effect of *AID *expression observed in the most *E. coli *selective systems [31]. In the wild-type strain, there is some prevalence of mutations due to putative non-transcribed strand deaminations (Table 4) suggesting the possibility that in our system the repair of uracil in the transcribed DNA strand is more efficient than in non-transcribed strand. Clearly, *hAIDSc *is targeted to both DNA strands in yeast, similar to somatic mutations in G-C bases during SHM [6,7,9,57-59]. It is important to mention that, under normal circumstances, there are no differences in DNA strand preferences between mutation spectra from the wild-type and *ung1 *strains [60].

Next, we examined whether the DNA context of mutations induced by AID in yeast is similar to SHM mutations in mammals or in *E. coli *expressing *hAID *(Table 5). DGYW and GYW motifs [7,8,40] were under-represented in mutations occurring spontaneous in wild-type or *ung1 *strains (Table 5, row 1–2) and were 2 – 5 fold over-represented in mutations induced by AID in yeast (Table 5, rows 3–4). Lists of mutation hotspots are shown in Table 6. Distributions of AID-induced mutation hotspots in the wild-type and *ung1 *strains are significantly different (Table 6, P = 0.003). The specificity of mutations in yeast correlates better with the hotspot motifs for SHM in mice than does the specificity of AID induced mutations in *E. coli*. Indeed, out of four comparisons, the indices of preference for mutation hotspot motifs in yeast were higher than in *E. coli *(compare rows 3–4 with rows 5–8; rows 10–11 with rows 5–8, Table 5). Some properties of mutations in yeast resemble the *in vitro *AID induced mutation spectrum [40]. For example, one CGYW/WRCG sequence which is not mutable in SHM [8] had a high mutability in the *ung1 *mutation spectrum (Table 6). It has been suggested that a mammalian DNA repair enzyme, perhaps the uracil-DNA glycosylase, efficiently repairs the lesion of CpG dinucleotides and thus eliminates mutations from CGYW/WRCG motifs *in vivo *[8]. We have found 5 GGYW and 3 TGYW hotspots (Table 6). Interestingly, no hotspots were found in AGYW motifs (Table 6), which are the most frequent hotspot motifs in mammalian immunoglobulin genes [47]. The lack of mutation hotspots at AGYW could not be attributed to its lesser prevalence because the number of AGYW, GGYW, and TGYW motifs in *CAN1 *was similar (results not shown). However, a general pattern of mutations in yeast is similar to the targeting of somatic mutation in DGYW/WRCH mutable motifs, which are highly specific for SHM in mammals. In control, no significant targeting of mutations to DGYW/WRCH mutable motifs was found for spontaneous mutations in wild type or *ung1 *yeast (P_W≤Wrandom _> 0.05, Table 5, rows 1–2).

We did not find a substantial number of A-T mutations, which typically comprise one-half of all SHM [47,58,61]. This result corresponds with earlier published results on the expression of AID in *E. coli *[24,36], in yeast [48], in murine fibroblasts [13], and in human hybridomas [35]. Apparently, additional components are required to model the full spectrum of SHM under conditions of AID expression in heterologous systems or in non-B cell tissues.

## Discussion

### Mutator efficiency and specificity of expression of *hAIDSc*

Yeast is a well-studied model eukaryotic organism used for various genetic studies. Yeast was used in this study to characterize the mutator effects of ectopic expression of human AID. The *CAN1 *reporter gene has been chosen because of numerous mutational studies [52,54] and a well-characterized transcription pattern [62-64]. The results are different from studies of AID effects in prokaryotic models and *in vitro *experiments. We observed mutations arising due to deamination occurring in both DNA strands. In *E.coli*, transcription enhances deamination of the non-transcribed DNA strand, which is exposed as single-stranded DNA during the elongation reaction, but not mutation of the transcribed DNA strand, which is likely to be protected by *E. coli *RNA polymerase [42,43,65]. The observed DNA strand targeting of mutations in *ung1 *yeast closely resembles targeting of somatic mutations in vertebrate immunoglobulin genes (Table 5). Interestingly, there was a significant strand bias of mutations in wild-type yeast toward the non-transcribed strand (Tables 4 and 5). A more efficient repair of the transcribed DNA strand is one possible explanation of this asymmetry. Preferential nucleotide excision repair of the transcribed strand is a well-known phenomenon [66]. The possibility of transcription-coupled repair of uracil bases in DNA has not yet been thoroughly studied.

Interestingly, a strand bias toward the non-transcribed DNA strand was found in *Ung*^-/- ^*Msh2*^-/- ^mouse (Table 5, row 8) [67]. The difference between the number of mutations in DGYW/WRCH sites versus all other G:C sites in wild-type and *Ung*^-/-^*Msh2*^-/- ^strains was statistically significant (Fisher exact test, P = 0.04). This may indicate that AID has a preference to the non-transcribed DNA strand as suggested earlier (see review [32]). An excessive DNA deamination of the non-transcribed DNA strand may be compensated by more efficient repair of this strand during the SHM phase 2 [67] causing approximately equal frequencies of mutations in both DNA strands (Table 5). More efficient repair of the non-transcribed strand is consistent with the idea of preferential targeting of the DNA polymerase η to the non-transcribed strand during SHM [9,25]. In general, the strand specificity of SHM in *Ung*^-/-^*Msh2*^-/- ^mouse is similar to AID-induced mutations in wild-type strains of yeast and *E. coli *(Table 5). Substantial differences between the observed targeting of AID to the mutable motifs in *Ung*^-/- ^*Msh2*^-/- ^and wild-type mouse (Table 5) are not consistent with a hypothesis that mutagenesis during the A:T-focused phase is nearly exclusively targeted to A:T bases [67,68]. It is possible that mutagenesis during this phase is targeted to both A:T and G:C bases with a preference to A:T bases and no preference to DGYW/WRCH mutable motifs, this is consistent with the observed mutational and context specificity of the DNA polymerase η *in vitro *[9,25,69], DGYW/WRCH-independent mutagenesis of G:C bases will cause erosion of a high initial DGYW/WRCH motif specificity observed in *Ung*^-/-^*Msh2*^-/- ^mouse (Table 5). There are also differences between strand specificity of *Ung*^-/-^*Msh2*^-/- ^mouse and AID-induced mutations in human fibroblasts (Table 5), this might be explained by some differences in AID targeting or transcription-associated repair of uracil between B-lymphocytes and fibroblasts. All these results suggested that a weak strand bias is an intrinsic property of SHM.

A significant difference between *in vitro *systems and our experiments was observed. AID catalyses multiple deaminations *in vitro *[40]. We detected 11 clones with multiple mutations (10 clones with two mutations and one clone with three mutations) and checked the number of mutations in the first and second halves of *CAN1*. If multiple mutations emerge as a result of independent events, half of the clones are expected to have mutations in different halves of *CAN1*. In six out of 11 clones mutations were located in different parts of *CAN1*, thus independent mutation events is the most likely explanation. In general, the specificity and distribution of mutations in yeast did not exhibit a pattern of multiple mutations that would have been consistent with postulated processive action of AID [40]. These results are consistent with a high frequency of rearranged immunoglobulin V genes with one somatic mutation (for example, [70]). Apparent non-processive action of AID *in vivo *may be explained by a competition for binding to the *CAN1 *DNA sequence between AID and other proteins participating in transcription, replication and/or repair. For example, it is known that replication factor A stimulates AID [71], while the specificity of AID *in vitro *was studied on DNA without any additional factors. Clearly, this requires additional investigation.

### Mechanisms of mutagenesis by AID

The mechanism of SHM initiated by AID may be as follows (see [23,24,27,28,67,72]). Deamination of cytosine in DNA leads to the formation of a mismatched U-G base pair. If left unrepaired, further rounds of replication of uracil-containing DNA will generate only transition type mutations, G-C to AT. Uracil removal by uracil-DNA glycosylase leads to an apyrimidinic (AP) site. The AP site may be bypassed by a specialized DNA polymerase and, being a non-coding lesion, could lead to a transition or transversion mutation. The AP site may also be incised by AP-endonuclease and then repaired by the short patch base excision repair (BER) with involvement of error prone DNA polymerases with generation of transitions and transversions (e.g., see [72]). This mechanism generates mutations at G-C pairs. In order to explain the high frequency of mutations during the short patch BER reaction, it should be postulated that the relatively accurate DNA polymerase β is substituted in B-cells by an error-prone polymerase. The candidate is DNA polymerase ι, which is expressed in Burkitt's lymphoma cell line BL-2 [73] and whose inactivation suppresses SHM in this line [74]. However, 129-derived strains of mice, lacking active polymerase η, are fully proficient in SHM [75]. The reason for this discrepancy in not established yet.

Another type of mutation, which comprise about 50% of all mutations during SHM, is a change at the A-T base pairs [47,58,61]. The explanation of the mutation origins in A-T base pair is based on several observations. It is known that mutations at A-T base pairs depend on mismatch repair components *MSH2, MSH6, EXO1 *and error-prone DNA polymerase η [9,29,67,68,70,72]. They are thought to be the result of error-prone bypass or repair of abasic site by error-prone polymerases, in particular, DNA polymerases η, ι and ζ [9,25,70,73,74,76,77] (reviewed in [28,72,78,79]). It is possible that they are generated in the following way. Initiation of mismatch repair of a G-U base pair leads to a gap. Gaps may also be generated by long patch BER. Repair of gaps with the involvement of error-prone DNA polymerases may lead to mutations distal to initial G-U pair [25,68]. Again, it should be postulated that gap repair is unusual in B-cell being inaccurate, since normally it is performed by highly accurate replicative DNA polymerases. The final feature of the current model of AID-initiated genetic modifications is that nicks and gaps, arising during DNA repair, stimulate recombination [16,48]. SHM in the *Ung1*^-/- ^mice is greatly biased in favour of transitions, since the pathway via apyrimidinic sites is blocked [26].

Mutations at A-T base pairs are absent in *Msh2*^-/-^*Ung1*^-/- ^mice [67]. Is it important to notice that Ung1 is not a major enzyme involved in the overall repair of G:U mismatches in mice, as suggested by small mutator phenotypes in the *Ung1*^-/- ^mice and the existence of the robust Smug1 glycosylase [80]. In B-cells, however, the Ung1 alone appears to be crucial for all genetic diversification processes [67,81]. Mutations at A-T base are not observed when AID is expressed in prokaryotes or in yeast [31,36,48], and this work. Therefore, current model systems only partially reconstruct SHM. Delicate balance of mismatch repair and activity of error-prone polymerases, specific for B cells, might be required for the full spectrum of SHM mutations [68]. Changes in the chromatin structure are necessary for SHM [82] and this additional level of regulation should be taken into account when considering different SHM models.

## Conclusion

In the present study, we have shown that expression of human *AID *is mutagenic in yeast and the mutagenic effect is one-two orders of magnitude higher in the *ung1 *strain. This observation suggests that the cause of the mutator effects is AID-driven DNA deamination. DNA sequence contexts of mutation hotspots coincide with DGYW/WRCH mutable motifs of somatic hypermutation, which is consistent with the DNA deamination model of SHM, suggesting that the intrinsic substrate specificity of AID itself is a primary determinant of mutational hotspots at G-C base pairs during SHM.

## Methods

### Construction of the expression vector

A new *hAID *gene was constructed using codons characteristic to highly expressed yeast genes. The DNA Builder program  and yeast codon usage data [83,84] was used to construct a DNA sequence encoding human AID, with the preferable yeast codons. The DNA corresponding to this sequence and encoding for the c-myc Tag at the C-terminus (*hAIDSc*) was custom-synthesized and cloned into *Bam*HI-*Sal*I cut pESC-LEU (Stratagene) expression vector by the McLab Company (San Francisco). In this construct, the deaminase genes were placed downstream of the strong, galactose-inducible *GAL1 *promoter. DNA sequencing analysis confirmed the complete sequence of the insert. Protein production was demonstrated by Western blot as described earlier [85], with one modification – the Western Breeze Kit (Invitrogen) was used for detection of the protein in yeast extracts.

### Yeast strains

For our experiments with the yeast vector expressing the deaminase genes we used yeast strain CG379-3-29RL (*MAT α ura3Δleu2-3,112 trp1-289 bik1::ura3-29RL his7-2 ade5-1 lys2-Tn5-13*) [48,86,87]. This strain allows concomitant measurement of mutation rates at several loci. These include a) the forward mutation rate at the *CAN1 *locus, where mutations reflect a variety of substitution, frameshift and more complex events; b) the rate of reversion of nonsense mutations: the *trp1-289 *(TAG [88]) and *ade5-1 *(TAA, [89]), where mutations reflect base substitutions in the nonsense codon as well as in suppressor genes encoding tRNAs; c) reversion of the *ura3-29 *missense mutation TCT which occurs via C-G to T-A transitions, C-G to C-A and C-G to G-C transversions [50]; d) reversion of the *his7-2 *mutant allele which occurs mainly via + 1 frameshifts in a homopolymeric AT run [49,90].

### Measurement of mutations rates

Mutation rates were analysed by fluctuation analysis [49,90]. Independent transformants of the wild-type and *ung1 *derivatives of our basic strain were grown in a complete minimal medium lacking leucine to select for the plasmid, and containing galactose instead of glucose, to induce the *hAIDSc *expression.

### Isolation and sequencing of *can1 *mutants

Yeast transformants patches originating from single colonies (64 per one plate) were replica-plated onto galactose-containing medium without leucine. After two days, they were replica-plated onto canavanine-containing medium to select for *can1 *mutants. After five days of incubation, one Can^r ^colony was picked from each streak and streaked onto canavanine-containing medium. Chromosomal DNA from cells originating from one colony of these *can1 *mutants was isolated using a Yeast DNA Extraction Kit (Epicentre). Subsequent PCR amplification and sequencing was performed as described earlier [91].

### In vivo and in vitro mutation spectra

Five *in vivo *and one *in vitro *mutation spectra, which have been described before [13,26,36,40,47] were used in this study. We consider that these large mutation spectra reflect intrinsic bias in mutation process. The compilation of somatic mutations in the VkOx transgene includes data derived from transgenic light chains with multiple copies of the transgene and from cells selected in gut Peyer's Patches (PP). The multiple copies are targeted in the same cell even when the light chain they encode is not part of the antigen binding antibody molecule. This implies that the majority of the mutations accumulated are unselected. In the case of PP derived cells, the selective pressure is multiple, therefore again, the common denominator of the biases observed would reflect the intrinsic biases [92,93].

### Statistical analyses

The Fisher exact test was used to compare frequencies of transitions and transversions. This test was also used to compare the number of mutations in DGYW/WRCH sites versus all other G-C sites in wild-type and *Ung*^-/-^*Msh2*^-/- ^strains of mouse. A Monte Carlo modification of the Pearson χ^2 ^test of spectra homogeneity [94] was used to compare mutation distributions along hotspot positions of the *CAN1 *sequence. Calculations were done using the COLLAPSE program [95]. Mutations in the *CAN1 *gene were detected using the phenotypic assay described above, however the full list of detectable positions in this gene is not known. We predicted these positions using the SIFT program with default parameters [96]. Mutation hotspots were defined using a threshold for the number of mutations at a site. The threshold is established by analyzing the frequency distribution derived from a mutation spectrum using the CLUSTERM program [97]. Briefly, this program decomposes a mutation spectrum into several homogeneous classes of sites, with each class approximated by a binomial distribution. Variations in mutation frequencies among sites of the same class are random by definition (mutation probability is the same for all sites within a class), but differences between classes are statistically significant. Each site has a probability P(C) to be assigned to class C. A class with the highest mutation frequency is called hotspot class. Sites with of P(C_hotspot_) ≥ 0.95 of being assigned to the hotspot class C_hotspot _are defined as hotspot sites. This approach ensures that the assignment is statistically significant and robust (see Rogozin et al. [98] for detailed discussion of this approach and problems associated with its application).

Nucleotide sequence features can be correlated with a mutation spectrum and the correlation can be tested for statistical significance. The significance of correlations between the distribution of mutable motifs and mutations along a target sequence was measured by a Monte Carlo procedure (the CONSEN program) [7]. This approach takes into account frequencies of substitutions for each nucleotide, the possibility of multiple mutations in a site, and context of the mutating sites. The Monte Carlo simulation was run with weighted sites, with the weight of a site defined as:



where M_j _is the number of mutations in site j. W_j _weights were summed for all sites in the analyzed sequence resulting in the total weight W. A distribution of total weights W_random _was calculated for 10,000 target sequences with randomly shuffled mutation spectra. Each of the resulting random mutation spectra contained the same number of mutations as the observed spectrum with the same distribution of mutations over randomly chosen sites. The distribution of W_random _was used to calculate probability P_W≤Wrandom_. This probability is equal to the fraction of random spectra in which W_random _is the same or greater than W. Small probability values (P_W≤Wrandom _≤ 0.05) indicate a significant correlation between a mutable motif and the mutation frequency [7,99].

## Authors' contributions

This work was designed by YIP, TAK and IBR. YIP and CF were involved in vector construction, Western blot analysis of proteins, mutation rates measurements and isolation of *can1 *mutants. VIM and LA performed PCR and DNA sequencing and were involved in analysis of mutations. IBR did statistical analysis of mutation spectra.

## Supplementary Material

Additional File 1 **Spectra of *can1 *mutations in yeast**. Types of nucleotides changes found are shown above the DNA sequence of the non-transcribed strand. A. *ung1 *strain, B. wild-type strain. Most of the mutations were single base pair substitutions. The multiple mutations found in the *CAN1 *are listed below. wild-type strain 452,C->T; 1728,T->G 123,A->G; 1392,G->A 123,A->G; 412,C->T 589,T->A; 1151,C->G 1485,G->T; 1486,C->A *ung1 *strain 553,C->A; 1633,C->T 1018,G->A; 1728,T->G 299,G->A; 1475,C->T 424,G->A; 980,G->A 572,T->C; 573,C->T 509,G->A; 516,G->A; 806,G->A Click here for file

## Figures and Tables

**Table 1:** Mutagenic effect of the *hAIDSc *expression in yeast.

Strain	Plasmid	Mutation rates*				
		Can^r ^× 10^-7^	Ade^+ ^× 10^-8^	Trp^+ ^× 10^-8^	Ura+ × 10^-8^	His^+ ^× 10^-8^

wild-type	vector	2.5	24	4.1	4.0	2.4
		1.2–6.5	21–34	1.3–14	2.2–6.6	1.9–2.9
						
	*hAIDSc*	19	72	24	4.0	2.3
		14–25	60–124	21–38	2.8–7.8	1.1–4.2
						

*ung1::hygB*	vector	13	210	134	23	3.0
		10–31	190–290	110–170	20–35	1.9–4.5
						
	*hAIDSc*	205	9700	5300	52	4.0
		170–220	7500–12600	4400–6600	43–77	2–-7.1

*Median mutation rates determined in 9–18 cultures. 95% confidence limits are shown below.

**Table 2:** DNA sequences changes in *can1 *mutants induced by expression of *hAIDSc*.

	Total	Observed sequence change		
		
Strain	Sequences with mutations	Single base substitution	Tandem double substitution	Frameshift
wild-type	67	68	1	2
*ung1*	59	64	1	0

**Table 3:** Types of base of substitutions found in *can1 *mutants induced by expression of *hAIDSc*.

Substitution	wild-type	*ung1*
G→A	19	37
C→T	37	24
G→T	2	0
C→A	4	1
G→C	0	0
C→G	2	0
A→G	2	2
T→C	1	1
A→C	0	0
T→G	1	1
A→T	0	0
T→A	2	0

**Table 4:** Differences in occurrence of transitions/transversion and mutations in two DNA strands of the *CAN1 *gene in wild-type and *ung1 *strains.

Spectra compared	wild-type	*ung1*	P_*fisher*_
Variables			
Transitions at G-C bases	56	61	
Transversions at G-C bases	8	1	0.032
Transcribed strand (G→A)	19	37	
Non-transcribed strand (C→T)	37	24	0.005

P_*fisher *_is the probability that a 2 × 2 contingency table is homogeneous as calculated using Fisher exact test.

**Table 5:** Mutations in different mutable motifs in different spectra.

Spectrum	Sequence motif (mutable positions are underlined)		Reference
		
	DGYW / WRCH	NGYW / WRCN	
Spontaneous *can1 *mutations in the wild-type yeast	0.5 / 0.5	0.7/0.5	[52]
Spontaneous *SUP4-o *mutations in *ung1 *yeast strain	0.9/1.1	0.9/1.1	[60]
*hAIDSc *in the *CAN1 *in wild-type yeast	***1.2 / 5.2***	***1.5 / 4.8***	This work
*hAIDSc *in the *CAN1 *in *ung1 *yeast	***2.0 / 1.8***	***3.2 / 1.6***	This work
SHM in V_κ_Ox1 in mouse	***3.5 / 2.5***	***3.4 / 2.5***	[47]
SHM in J_H_4 region in wild-type mouse	***4.4 / 3.2***	***4.4 / 3.2***	[26]
SHM in J_H_4 region in *Ung1*^-/- ^mouse	***4.5 / 3.6***	***4.5 / 3.6***	[26]
SHM in J_H_4 region in *Ung1*^-/- ^*Msh2*^-/- ^mouse	***3.3 / 5.0***	***3.2 / 5.0***	[67]
AID in *GFP *in human fibroblasts	***7.6 / 4.8***	***7.1 / 4.1***	[13]
AID in *sacB *in *E.coli*	***2.3 / 3.7***	***1.6 / 4.6***	[36]
AID in *lacZ in vitro*	***-- / 2.1***	***-- / 2.2***	[40]

The values listed represent the fold increase in occurrence of mutations at mutable motifs above the average occurrence of mutations at other G-C sites. Number of mutations in mutable motifs was calculated for the underlined bases. Bold italicized values represent a statistically significant correlation (P ≤ 0.05) between a mutable motif and the distribution of mutations, as revealed by using a Monte Carlo procedure [7].

**Table 6:** Base substitution hotspots and mutable motifs.

Number of substitutions				
Position	Sequence	Wild-type	*ung1*	NGYW/WRCN variant (N = A/T/C/G)

238	G**TA C**AGA	**4**	2	TGYW
268	A**AG C**AAA	**7**	2	TGYW
299	GTG **G TA**C	1	**4**	GGYW
424	GGT G AAA	0	**3**	-
612	ATG G AAT	0	**3**	-
896	AAG **G TA**C	**3**	1	GGYW
980	TCC **G TA**T	2	**6**	CGYW
1166	C**TG C**CGC	**4**	0	GGYW
1392	A**TG G**TTA	**3**	2	GGYW
1426	A**TG C**AAG	1	**4**	TGYW
1486	A**TG C**CCG	**3**	0	GGYW

The CLUSTERM program [97] was used for hotspot analysis. The hotspot threshold value is three mutations for both spectra. Hotspot motifs are shown in Bold and enlarged font. The hotspot base is underlined.
