# Supplementary material for: Expression of human AID in yeast induces mutations in context similar to the context of somatic hypermutation at G-C pairs in immunoglobulin genes
Source: BMC Immunol. 2005 Jun 10;6:10. doi: 10.1186/1471-2172-6-10 (PMC1180437; doi:10.1186/1471-2172-6-10)
Supplement: Additional File 1 — Spectra of can1 mutations in yeast. Types of nucleotides changes found are shown above the DNA sequence of the non-transcribed strand. A. ung1 strain, B. wild-type strain. Most of the mutations were single base pair substitutions. The multiple mutations found in the CAN1 are listed below. wild-type strain 452,C->T; 1728,T->G 123,A->G; 1392,G->A 123,A->G; 412,C->T 589,T->A; 1151,C->G 1485,G->T; 1486,C->A ung1 strain 553,C->A; 1633,C->T 1018,G->A; 1728,T->G 299,G->A; 1475,C->T 424,G->A; 980,G->A 572,T->C; 573,C->T 509,G->A; 516,G->A; 806,G->A [file 1471-2172-6-10-S1.pdf]

A.

```
ATGACAAATTCAAAAGAAGACGCCGACATAGAGGAGAAGCATATGTACAATGAGCCGGTCACAACCCTCTTTCACGACGTTGAAGCTTCACAAACACACC
         10        20        30        40        50        60        70        80        90       100

                      G
                      G
ACAGACGTGGGTCAATACCATTAAAAGATGAGAAAAGTAAAGAATTGTATCCATTGCGCTCTTTCCCGACGAGAGTAAATGGCGAGGATACGTTCTCTAT
        110       120       130       140       150       160       170       180       190       200

                                                                                                  A
                                                                                                  A
                                     T                             T                              A
                                     T                             T                              A
GGAGGATGGCATAGGTGATGAAGATGAAGGAGAAGTACAGAACGCTGAAGTGAAGAGAGAGCTTAAGCAAAGACATATTGGTATGATTGCCCTTGGTGGT
        210       220       230       240       250       260       270       280       290       300

       A  T                                                    A
ACTATTGGTACAGGTCTTTTCATTGGTTTATCCACACCTCTGACCAACGCCGGCCCAGTGGGCGCTCTTATATCATATTTATTTATGGGTTCTTTGGCAT
        310       320       330       340       350       360       370       380       390       400

                       A
                       A
                       A                                                                        A
ATTCTGTCACGCAGTCCTTGGGTGAAATGGCTACATTCATCCCTGTTACATCCTCTTTCACAGTGTTCTCACAAAGATTCCTTTCTCCAGCATTTGGTGC
        410       420       430       440       450       460       470       480       490       500

        A            A
        A      A     A                          A                  CT  T
GGCCAATGGTTACATGTATTGGTTTTCTTGGGCAATCACTTTTGCCCTGGAACTTAGTGTAGTTGGCCAAGTCATTCAATTTTGGACGTACAAAGTTCCA
        510       520       530       540       550       560       570       580       590       600

           A
           A
           A                                           T                             A
CTGGCGGCATGGATTAGTATTTTTTGGGTAATTATCACAATAATGAACTTGTTCCCTGTCAAATATTACGGTGAATTCGAGTTCTGGGTCGCTTCCATCA
        610       620       630       640       650       660       670       680       690       700

                                                                                       A
AAGTTTTAGCCATTATCGGGTTTCTAATATACTGTTTTTGTATGGTTTGTGGTGCTGGGGTTACCGGCCCAGTTGGATTCCGTTATTGGAGAAACCCAGG
        710       720       730       740       750       760       770       780       790       800

     A  A                                                                                      A  T
TGCCTGGGGTCCAGGTATAATATCTAAGGATAAAAACGAAGGGAGGTTCTTAGGTTGGGTTTCCTCTTTGATTAACGCTGCCTTCACATTTCAAGGTACT
        810       820       830       840       850       860       870       880       890       900

                                                                               A
                                                                               A
                                                                               A
                                                                               A
                                                                              TA
                                                                              TA
GAACTAGTTGGTATCACTGCTGGTGAAGCTGCAAACCCCAGAAAATCCGTTCCAAGAGCCATCAAAAAAGTTGTTTTCCGTATCTTAACCTTCTACATTG
        910       920       930       940       950       960       970       980       990      1000

                 A
   T             A
GCTCTCTATTATTCATTGGACTTTTAGTTCCATACAATGACCCTAAACTAACACAATCTACTTCCTACGTTTCTACTTCTCCCTTTATTATTGCTATTGA
       1010      1020      1030      1040      1050      1060      1070      1080      1090      1100

                                                              T
                                                              T                             T  A
GAACTCTGGTACAAAGGTTTTGCCACATATCTTCAACGCTGTTATCTTAACAACCATTATTTCTGCCGCAAATTCAAATATTTACGTTGGTTCCCGTATT
       1110      1120      1130      1140      1150      1160      1170      1180      1190      1200

             T
TTATTTGGTCTATCAAAGAACAAGTTGGCTCCTAAATTCCTGTCAAGGACCACCAAAGGTGGTGTTCCATACATTGCAGTTTTCGTTACTGCTGCATTTG
       1210      1220      1230      1240      1250      1260      1270      1280      1290      1300

                                                                              A            A
                                                                              A            A
GCGCTTTGGCTTACATGGAGACATCTACTGGTGGTGACAAAGTTTTCGAATGGCTATTAAATATCACTGGTGTTGCAGGCTTTTTTGCATGGTTATTTAT
       1310      1320      1330      1340      1350      1360      1370      1380      1390      1400

                         T
                         T
                         T
                         T                                                T
CTCAATCTCGCACATCAGATTTATGCAAGCTTTGAAATACCGTGGCATCTCTCGTGACGAGTTACCATTTAAAGCTAAATTAATGCCCGGCTTGGCTTAT
       1410      1420      1430      1440      1450      1460      1470      1480      1490      1500
```

```
                                    T
                                    T
TATGCGGCCACATTTATGACGATCATTATCATTATTCAAGGTTTCACGGCTTTTGCACCAAAATTCAATGGTGTTAGCTTTGCTGCCGCCTATATCTCTG
      1510      1520      1530      1540      1550      1560      1570      1580      1590      1600

                      A         T
TTTTCCTGTTCTTAGCTGTTTGGATCTTATTTCAATGCATATTCAGATGCAGATTTATTTGGAAGATTGGAGATGTCGACATCGATTCCGATAGAAGAGA
      1610      1620      1630      1640      1650      1660      1670      1680      1690      1700

                           G
CATTGAGGCAATTGTATGGGAAGATCATGAACCAAAGACTTTTTGGGACAAATTTTGGAATGTTGTAGCATAG
      1710      1720      1730      1740      1750      1760      1770
```

B.

```
                                                                                          T
                                                                                          T
ATGACAAATTCAAAAGAAGACGCCGACATAGAGGAGAAGCATATGTACAATGAGCCGGTCACAACCCTCTTTCACGACGTTGAAGCTTCACAAACACACC
        10        20        30        40        50        60        70        80        90       100

                      G
                      G
ACAGACGTGGGTCAATACCATTAAAAGATGAGAAAAGTAAAGAATTGTATCCATTGCGCTCTTTCCCGACGAGAGTAAATGGCGAGGATACGTTCTCTAT
       110       120       130       140       150       160       170       180       190       200

                                                                   T
                                                                   T
                                                                   T
                                     T                             T
                                     T                             T
                                     T                             T
                                     T                  A          T                              A
GGAGGATGGCATAGGTGATGAAGATGAAGGAGAAGTACAGAACGCTGAAGTGAAGAGAGAGCTTAAGCAAAGACATATTGGTATGATTGCCCTTGGTGGT
       210       220       230       240       250       260       270       280       290       300

          T
          T                                                     T
ACTATTGGTACAGGTCTTTTCATTGGTTTATCCACACCTCTGACCAACGCCGGCCCAGTGGGCGCTCTTATATCATATTTATTTATGGGTTCTTTGGCAT
       310       320       330       340       350       360       370       380       390       400

           T                                       T
           T                                    G  T                   TT
ATTCTGTCACGCAGTCCTTGGGTGAAATGGCTACATTCATCCCTGTTACATCCTCTTTCACAGTGTTCTCACAAAGATTCCTTTCTCCAGCATTTGGTGC
       410       420       430       440       450       460       470       480       490       500

        A                                           T                                   A
GGCCAATGGTTACATGTATTGGTTTTCTTGGGCAATCACTTTTGCCCTGGAACTTAGTGTAGTTGGCCAAGTCATTCAATTTTGGACGTACAAAGTTCCA
       510       520       530       540       550       560       570       580       590       600

                         AA
CTGGCGGCATGGATTAGTATTTTTTGGGTAATTATCACAATAATGAACTTGTTCCCTGTCAAATATTACGGTGAATTCGAGTTCTGGGTCGCTTCCATCA
       610       620       630       640       650       660       670       680       690       700

AAGTTTTAGCCATTATCGGGTTTCTAATATACTGTTTTTGTATGGTTTGTGGTGCTGGGGTTACCGGCCCAGTTGGATTCCGTTATTGGAGAAACCCAGG
       710       720       730       740       750       760       770       780       790       800

                                                                                               A
                                                                                               A
                                                                                           A   A  T
TGCCTGGGGTCCAGGTATAATATCTAAGGATAAAAACGAAGGGAGGTTCTTAGGTTGGGTTTCCTCTTTGATTAACGCTGCCTTCACATTTCAAGGTACT
       810       820       830       840       850       860       870       880       890       900

                                                                               A
                        A  A         T                    T                   TA
GAACTAGTTGGTATCACTGCTGGTGAAGCTGCAAACCCCAGAAAATCCGTTCCAAGAGCCATCAAAAAAGTTGTTTTCCGTATCTTAACCTTCTACATTG
       910       920       930       940       950       960       970       980       990      1000

                 A                A                  T
GCTCTCTATTATTCATTGGACTTTTAGTTCCATACAATGACCCTAAACTAACACAATCTACTTCCTACGTTTCTACTTCTCCCTTTATTATTGCTATTGA
      1010      1020      1030      1040      1050      1060      1070      1080      1090      1100

                                                                 T
                                                                 T
                                                                 T                          T
                                                  G              T                          T  AA
GAACTCTGGTACAAAGGTTTTGCCACATATCTTCAACGCTGTTATCTTAACAACCATTATTTCTGCCGCAAATTCAAATATTTACGTTGGTTCCCGTATT
      1110      1120      1130      1140      1150      1160      1170      1180      1190      1200
```

```
          C                                                  A
TTATTTGGTCTATCAAAGAACAAGTTGGCTCCTAAATTCCTGTCAAGGACCACCAAAGGTGGTGTTCCATACATTGCAGTTTTCGTTACTGCTGCATTTG
      1210      1220      1230      1240      1250      1260      1270      1280      1290      1300

                                                                                           A
                                                                                           A
                                                                              T            A
GCGCTTTGGCTTACATGGAGACATCTACTGGTGGTGACAAAGTTTTCGAATGGCTATTAAATATCACTGGTGTTGCAGGCTTTTTTGCATGGTTATTTAT
      1310      1320      1330      1340      1350      1360      1370      1380      1390      1400

                                                                                     T
                                                                                     T
  A                      T                                                          TA
CTCAATCTCGCACATCAGATTTATGCAAGCTTTGAAATACCGTGGCATCTCTCGTGACGAGTTACCATTTAAAGCTAAATTAATGCCCGGCTTGGCTTAT
      1410      1420      1430      1440      1450      1460      1470      1480      1490      1500

TATGCGGCCACATTTATGACGATCATTATCATTATTCAAGGTTTCACGGCTTTTGCACCAAAATTCAATGGTGTTAGCTTTGCTGCCGCCTATATCTCTG
      1510      1520      1530      1540      1550      1560      1570      1580      1590      1600

                     A
TTTTCCTGTTCTTAGCTGTTTGGATCTTATTTCAATGCATATTCAGATGCAGATTTATTTGGAAGATTGGAGATGTCGACATCGATTCCGATAGAAGAGA
      1610      1620      1630      1640      1650      1660      1670      1680      1690      1700

                           G
CATTGAGGCAATTGTATGGGAAGATCATGAACCAAAGACTTTTTGGGACAAATTTTGGAATGTTGTAGCATAG
      1710      1720      1730      1740      1750      1760      1770
```
